# American Orthopedic Association

**Published:** 1888-10

**Authors:** 


					﻿AMERICAN ORTHOPEDIC ASSOCIATION.
Second Annual Meeting, held at Wash-
ington, D. C., September 18, 19, and 20.
First Day—Morning Session.
The Association met, and was called to
order at ten o’clock by the President, Dr.
Newton M. Shaffer, of New York.
The Secretary, Dr. Lewis Hall Sayre,
of New York, read the minutes of the pre-
liminary meeting, which were approved.
Professor V. P. Gibney, of New York,
read a paper on “ The Treatment of Club-
foot by means of the Thomas Wrench, or
T. T.,” and presented an apparatus.
First Day—Afternoon Session.
Dr. H. Hodgen, of St. Louis, read a
paper on “Morton’s Operation for the Im-
mediate Reduction of the Club-foot,” and
reported five cases of congenital equino-
varus, three of which were double.
His deductions were : 1. Tenotomy for
equinus should be made first and the de-
formity at once reduced. 2. Immediate
reduction of the deformity is the proper
procedure. It saves time, pain, and' the
correction is more certain. 3. It can be
used in all cases with safety, but has no
advantage over hand-pressure of the sur-
geon in young subjects when the tissues are
soft. 4. There is no need of preparatory
treatment of the tissues by poulticing. 5.
The force should be'applied inwards, down-
wards, and slightly forwards, until the tissues
are ready to give way. 6. Plaster dressing,
applied with the foot in position of varus,
and allowed to remain for two months. 7.
The bursae will have nearly disappeared
when the cast is removed, and they are not
as a rule tender. 8. The use of a club-
foot shoe for some time, in which pressure
can be made in the direction of the crush-
ing force. 9. A marked rise of tempera-
ture is not to be expected, and when this
and pain are present one must look to the
plaster as the cause in a large per centage
of these cases.
This was followed by a paper entitled
“A Practical Point in the Treatment of
Pott’s Disease of the Spine,” by Dr. A. B.
Judson, of New York.
He believes that in the unsatisfactory state
of the treatment of Pott’s disease it would
be well to judge of the efficiency of mechan-
ical treatment by the impression made by
the apparatus on the skin, which should be
kept dry, covering the projection. The
object of treatment is three fold—to fix
the diseased bones ; to transfer pressure
from the bodies to the processes, and to
reduce the deformity. If the skin near the
summit of the projection becomes thickened
and callous, which it does without discom-
fort if the pressure is carefully increased
from time to time, it is to be considered
that the apparatus is acting as it ought to,
and when the great pressure is made com-
patible with the integrity and comfort of
the skin, the apparatus has reached the limit
of its efficiency. This rule is less applica-
ble to the treatment by suspension and the
plaster-of-Paris jacket. In the hands of the
general practitioner plaster of Paris gives
relief to many cases which would otherwise
have no mechanical treatment. But the
orthopedic surgeon can do better by the
use of tractable steel, modified to fit the
varying needs of the patient with the me-
chanical skill which belongs to practition-
ers of that class.
Dr. E. H. Bradford, of Boston, present-
ed an “Analysis of Treatment of Seventy
Cases of Club-foot.”
He said the treatment necessarily re-
quires :
1.	The rectification of the misplaced
bones.
2.	Retention in a normal position until
the abnormal facet of the astragalus renders
the pressure of a new position normal.
The following questions in regard to
treatment are at present unsettled :
a.	In what cases is it advisable to cor-
rect and treat by mechanical means alone,
without the aid of any operative interfer-
ence, even tenotomy ?
b.	When is tenotomy advisable ?
c.	Can severe cases be entirely corrected
and permanently cured without tarsal re-
section ?
d.	Is tarsal resection or osteotomy a jus-
tifiable operation ?
His conclusions were:
1.	That the cases of infantile club-foot
can, as a rule, be thoroughly and efficiently
treated without tenotomy by mechanical
correction and mechanical retention alone.
2.	That in older cases tenotomy aids
the correction, and is not injurious in the
permanent result.
3.	That resistant cases of the severest
type can be corrected without tarsal oste-
otomy.
4.	That in some cases of resistant club-
foot tarsal osteotomy is needed for perfect
rectification, and is not only justifiable, but
may be indicated in exceptional instances.
Dr. G. W. Ryan, of Cincinnati, reported
a case of reflex valgus.
Second Day—Morning Session.
Dr. Royal Whitman, of Boston, read a
paper entitled “Observations on Seventy-
five Cases of Flat-foot, with Particular
Reference to Treatment.”
Clinically, flat-foot is an affection which,
in the majority of cases, occurs independent
of local or constitutional disease. The di-
rect cause is overweight or overwork for
feet subjected to mechanical disadvantages.
These are (i) the common habit of turning
on the toes in standing and walking, an at-
titude which brings an increased and contin-
ual strain on the weakest part of the foot.
(2) Weakness of muscles from disease, and
deformities caused by improper shoes. Flat-
foot being essentially a dislocation should
be treated as other dislocations, by reposi-
tion and retention in normal positions, until
by exercise and avoidance of faulty posi-
tion the muscles may regain their normal
strength. In ordinary cases such retention
can only be accomplished by some form of
mechanical support. A brace should be
constructed on principles somewhat as fol-
lows : It should be inelastic, light, and
comfortable ; should fit the foot perfectly,
and should not interfere with its normal
movements, or the action of its muscles. A
steel brace designed on these principles was
shown. The paper was illustrated by casts
and diagrams.
Dr. John Ridlon, of New York, exhib-
ited a wrench which he used for the same
purpose as that exhibited by Professor Gib-
ney, it being modified from a “utility”
wrench. It differed from Thomas’ and Gib-
ney’s modification of Thomas’ wrench in
that the grip was taken by sliding the lower
grip-bar towards the upper simply by man-
ual pressure. When in position it was held
by a cog-and-spring arrangement which
could instantly be released by pressing a
trigger. The grip-bars were straight, in-
stead of curving towards each other as in
the Gibney instrument. This was an ad-
vantage when the instrument was used as
an osteoclast in cases of badly united Colles’
fracture of the radius. As an instrument
for cases of club-foot the author did not
think it had any special advantages over
Thomas’ wrench.
Dr. Ap. Morgan Vance, of Louisville,
read a clinical report of eight cases of
femoral osteotomy for the correction of de-
formity resulting from hip-joint disease in
five of the cases, and knee-joint disease in
the remaining three. He advocated the
subcutaneous method as being better than
the other operation by open wound, stating
that he would break a man’s femur sub-
cutaneously with as little misgivings as to
the outcome as he would divide his tendon
Achilles. All of the cases reported had
practically reached adult life, the youngest
being 14J years of age, the oldest 32.
Second Day—Afternoon Session.
The President exhibited Colin’s osteo-
clast to the Association.
Dr. Henry L. Taylor, of New York,
read a paper on “ Senile Coxitis.”
By senile coxitis he understood a rheu-
matic affection of the hip-joint, occurring
in old people, and characterized by pain,
stiffness, muscular spasm, and disability,
but never showing a tendency to suppura-
tion. It may or may not be associated
with similar trouble in other joints. The
point he makes is that some of the cases
which have resisted the ordinary medic-
inal and dietetic treatment, and in whom
the hip-joint disorder is of such a grade as
to practically incapacitate them from loco-
motion, may still be very much benefited
and the comfortable use of the limb re-
stored by resorting to mechanical treat-
ment.
He cited two cases in females about 57
years old, suffering from senile coxitis of
disabling grade ; in one, the disease was
non-articular; in the other, several joints
were affected in addition to the hip. In
the first, although the disease has lasted
eight years, hip trouble had never before
been suspected, as the pain and lameness
were referred to the knee. These patients
were put to bed from four to six weeks,
with the long counter-extension hip-splint
and sixteen to eighteen pounds extra
weight, and were then allowed to get up
on the jointed supporting splint with per-
ineal strap, which protection was kept up
for about two years, with subsidence of
active symptoms in the joint and restora-
tion to comfortable, unaided locomotion.
So far as heard from they continued well.
Dr. Benjamin Lee, of Philadelphia,
read a paper on “ Haematoma Oris as a
Sign of Injury to the Spine in the Superior,
Cervical Region.”
His conclusions were: (i) That this
pathological condition is idiopathic, never
traumatic. He would not deny the exist-
ence of such a condition. It has a facies
and history of its own. The same amount
of violence would probably produce an
extravasation of blood in the tissues of the
ear in an insane person, but that would not
be the “ insane ear.” (2) That the local
tissue-change which makes an extravasa-
tion possible is dependent upon a degen-
erative alteration in the nerve centre which
controls the circulatory apparatus of the
auricle, involving, or not, the larger arterial
branches.
This paper was followed by one on “Os-
teotomy for Anterior Tibial Curves,” by Dr.
De Forrest Willard, of Philadelphia.
Rickets, although a constitutional disease
dependent upon mal-assimilation and mal-
nutrition, yet exhibits its worst phases in
deformities of the bones.
Anterior tibial curves are among the
most frequent of these distortions.
Treatment: (1) Manual straightening
and the employment of suitable apparatus.
This may be successful in young children
with the bones still springy, but in all other
cases it will utterly fail, and time and money
will be wasted.
Braces are useful in laterally bowed legs,
but not in anterior curves.
2. Forcible fracture:	manual; b,
instrumental.
a. Manual fracture. A strong surgeon
can usually break the bones of a child
under 3 years of age, especially if the knee
be brought into use as a fulcrum, but the
point of fracture is uncertain. The result
is a simple fracture.
b. Osteoclasis. The osteoclast applied
to the thigh is a definite and comparatively
certain and exact instrument in determin-
ing the point of fracture, but in the leg the
sharp tibial edge renders the employment
of much force a dangerous procedure as
regards the life of the soft parts.
3.	Osteotomy :	<z, simple ; • cunei-
form.
a.	Simple section of the bone with the
osteotone, although it produces a compound
fracture, is, practically, almost subcutane-
ous, and if done with thorough asepsis, is
rarely followed by suppuration. It is ap-
plicable to all except the very worst cases
of deformity, since even sharp angles of
bone are speedily inclosed in the callus.
Suturing of the divided bone is rarely nec-
essary.
b.	Wedge-shaped osteotomy is applicable
to severer grades of deformity. This op-
eration is much more likely to be followed
by suppuration, but with strict asepsis such
an accident is rare. Plaster of Paris fur-
nishes the most convenient form of after-
splint.
Osteotomy offers the simplest, surest, and
most satisfactory results in anterior tibial
curves.
Illustrative cases were described and the
details of the antiseptic operation fully
enumerated.
Dr. A. J. Steele, of St. Louis, read a
paper on “Two Knee-joint Excisions.”
In these cases he had done excision of
the knee, the first in a lad with chronic
fungoid and suppurative joint-disease
which had been going on from early child-
hood. The second in a young woman with
right-angled anchylosis of nine years’
standing, resulting from injury, and doubt-
less subsequent to acute synovitis. In the
first case the joint was rapidly going on to
destruction and the constitution breaking
down ; in the second, the inconvenience in
walking prompted measures for relief.
It was hoped arthrectomy alone might
afford the required relief in Case I, but on
exposing the joint the serious bone in-
volvement prompted its removal. Anti-
sepsis was used, all diseased tissue removed,
including the patella, the bones pinned
together with steel nails, and permanent
dressings applied. Subsequent oozing
caused change of dressing to a bracketed
plaster-of-Paris splint, with suspension.
Union was rapid and constitutional im-
provement prompt. At the present time,
three months since the operation, the boy
is in robust health and about on crutches,
wound healed, bones firm, and limb straight.
The removed eroded bones, the pins used,
and photograph of limb, were shown.
In Case II strict antisepsis was not ob-
served. The joint was removed en bloc, leg
straightened and bones doweled together
with four needles, the ordinary crotchet,
a. bracketed wire-splint applied, and the
leg suspended ; no untoward symptoms oc-
curred and little or no pus found. One
bone-pin was removed about the fourth
week, the other one breaking off in the
bone. It was expected this piece left in
would soften and be absorbed. For a time
it seemed to cause no irritation, but after
three months it caused suppuration, came
to the surface and was picked out, being
quite eroded. At the present time, eight
months since the operation, the patient is
walking, usually without even the assistance
of a cane, on a three inch patten, and with
scarcely a limp.
Dr. W. R. Whitehead, of Denver, read
a lengthy paper entitled “ Remarks on the
Operative and Mechanical Treatment of
some Joint-Diseases and Injuries, with
Special Reference to the Hip, Knee and
Elbow Joints, with Illustrative Cases.”
Dr. R. W. Lovett, of Boston, read a
paper on the “Treatment of Hip-Joint
Disease by Fixation and Extension, with
the Exhibition of a New Splint.”
He said American traction splints are
criticized by English surgeons on the
ground that they afford imperfect fixation.
With a view of determining how much fixa-
tion traction alone affords, some experi-
ments were made upon a healthy child. It
was found that with four pounds traction,
exerted by means of a hip-splint with one
perineal band, in walking 350 motion at
the hip-joint took place. With the highest
endurable traction (8 lbs.) 150 of motion
took place. Evidently traction alone can-
not afford fixation to a diseased joint. It
does not seem likely, either, that it sepa-
rates the joint surfaces, inasmuch as in the
case experimented on the power of the
thigh muscles was roughly estimated to be
36 pounds. With all this force, when set
in muscular spasm, they would pull the
head of the bone against the acetabulum
with more than that power, and the antag-
onizing force is only eight pounds at most.
Experiments as to distraction upon the
cadaver do not reproduce the clinical conn
ditions, inasmuch as muscular spasms, the
most important factor of all, is left out of
account.
A hip-splint providing fixation as well as
traction was shown, being merely a slightly
modified combination of the English
Thomas splint with the American traction
splint. Its best use is in bad cases where
any joint-motion does harm, and in cases
where deformity is present, inasmuch as it
is possible to correct the deformity while
the child goes about on crutches.
Dr. Samuel Ketch, of New York, read
a paper on “ Lateral Curvature and its
Early Treatment.”
The Association then elected the follow-
ing officers for 1889:
President—E. H. Bradford, Boston.
First Vice-President—Benjamin Lee,
Philadelphia.
Second Vice-President—V. P. Gibney,
New York.
Secretary and Treasurer—R. W. Lovett,
Boston.
Next place of meeting, Boston, in Sep-
tember, j 889.
AMERICAN RHINOLOGICAL
ASSOCIATION.
First Day—Morning Session.
The sixth annual meeting was held
at Cincinnati, Ohio, September 12,	13,
and 14.
The Association was called to order by
the President, at ten o’clock.
Dr. A. B. Thrasher, of’ Cincinnati, de-
livered an address of welcome, after which
the President delivered the annual ad-
dress, in which he recalled the progress
that has been made in rhinology.
Dr. J. E. Schadle, of St. Paul, Minne-
sota, read a report of “A Case of Chorea of
the Soft Palate, caused by the Hypertrophy
and Hypersesthesia of the Mucous Mem-
brane Covering both Inferior Turbinated
Bodies.”
He said some forms of headache and of
spasmodic asthma are, in many instances,
the outgrowth of a nasal polypus, a turbi-
nated thickening, or a septal deformity.
These reflex neurotic disturbances disap-
pear after appropriate measures of treat-
ment have been directed towards the re-
moval of their cause.
A patient was examined by Dr. Shadle
on April 16, 1888. She suffered from con-
stipation and flatulency quite frequently.
The heart’s action was more or less excited;
the heart beat tumultuously when ascend-
ing a flight of stairs, or some other eleva-
tion. She complained of marked obstruct-
ed nose-breathing; her voice was impaired,
especially when singing. The principal
difficulty for which she was sent to him was
the spasmodic movement of the velum
palati.
Faucial examination revealed distinct
rythmical choreiform movements of the
velum palati, accompanied by a peculiar
clicking sound, distinctly audible for a dis-
tance of twelve or fifteen feet from the pa-
tient. Anterior rhinoscopic examination
revealed chronic hypertrophy of the inferior
turbinated bones, more especially the mid-
dle one, whose redundant tissue caused
pressure on the septum and produced ob-
struction of the middle meatus.
Treatment was mainly surgical. Snaring
was impracticable. By the use of Cohen’s
post-nasal cutting forceps and the electro-
cautery, he reduced the growth entirely,
the procedure being followed by cessation
of the choreiform movements.
Afternoon Session.
Dr. Thos. F. Rumbold, of St. Louis,
Missouri, read a paper on “ The Etiology
and Pathology of Nasal Diseases.” The
etiology of a disease is of the greatest im-
port. A perfect understanding of its causes
and its mechanism is the goal of the path-
ologist. As regards heredity in the causa-
tion of rhinitis, he had long been convinced
that such a theory was erroneous. The
histological facts concerning cell-life would
prove a successful contravention of the
theory of heredity.
Second Day—Morning Session.
A paper entitled “ The Relation of Nasal
Diseases to Other Affections, Including the
Brain and Nervous System,” was read by
Dr. John North, of Keokuk, Iowa.
There is no organ, he said, in the entire
body that has been so neglected as the
nose. Obscure and obstinate diseases have
not yielded to treatment because it has
been directed to the effect of nasal re-
flex troubles and not to the nose, the cause
of the trouble.
He claimed that in 1886 he had shown
almost conclusively that certain cases of
neurasthenia were the result of chronic naso-
pharyngeal catarrh, and reported several
cases to substantiate his argument, and
which yielded to treatment applied to the
naso-pharynx.
Dr. Thos. F. Rumbold read a paper on
“ The Effect of Nasal Inflammation on the
Mind.” A normal condition of the mind
depends upon a normal supply of blood to
the normal brain. If an acute inflamma-
tion in the nasal passages is accompanied
by mental manifestations, mental incapacity
of a more severe character must accompany
a chonic rhinitis.
A paper was presented on “ The Etiolo-
gy and Pathology of Acute Catarrh,” by
Dr. J. G. Carpenter, of Stanford, Ken-
tucky.
Among the most prominent causes, he
mentioned occupations attended with dust,
smoke, excessive moisture, or dry, dusty
atmosphere, going about insufficiently clad,
the evil results of ordinary slippers, etc.
Pathologically, acute catarrh is divided
into three stages: (i) The dry stage, at-
tended with hypersemia, redness, heat,
tumefaction, and pain of mucous mem-
brane, the latter presenting a red, dry, or
dark-red appearance. (2) Moist stage,
supplementing the first; there is transuda-
tion of serum, diapedesis of the white cor-
puscles into the connective tissue, cell-
proliferation, and organization of lymph.
(3) The presence of pathological condi-
tions, with denudation of the epithelium
and the presence of a raw surface, and the
excessive mucous secretion is changed to
a muco-purulent or purulent one.
Afternoon Session.
Dr. R. S. Knode, of Fort Wayne, Indi-
ana, read a paper on “ Intra-Nasal Obstruc-
tion.”
He said, of all the mucous surfaces that
of the nose is perhaps the most sensitive
to irritating gases, vapors, and dust of dif-
ferent kinds, as well as sudden changes of
atmosphere. Intra-nasal obstructions may
be temporary or permanent, partial or com-
plete, owing to the extent of inflammation
or injury that produces them. Temporary
occlusion is usually the result of some one
of the forms of acute rhinitis. From severe
attacks, or from the effects of repeated
colds, the membrane may be found over-
whelmed with plastic material, with a loss
of nerve power, a pale, flabby, wrinkled
condition, somewhat resembling polypus,
but more sensitive to the touch, and com-
pletely filling up both cavities of the nose.
In this condition he had found nothing to
act as promptly as to first use a solution of
cocaine, applied with a probe and cotton,
and followed with mildly stimulating solu-
tions of chromic acid, ten grains to one
ounce, after which the vaseline treatment
should be used. If stenosis should return,
a second application of the acid may be
made, or slippery-elm plugs, slightly moist-
ened with a weak preparation of cocaine
and listerine.
Dr. E. G. Kegley, of Cedar Rapids,
Iowa, reported “A Case of Enlarged Ton-
sils, with Peculiar Symptoms, Relieved by
the Galvano-Cautery Snare.”
The patient was 43 years of age male ;
first consulted him in January, 1888,
with regard to a trouble which the
patient termed “ drooling.” He pre-
sented the appearance of one suffering
from hay-fever. His eyes were red
and swollen, with excessive lachryma-
tion, constant flowing and wiping of the
nose, and spitting of clear fluid. Examina-
tion revealed the nasal mucous membrane
highly congested, and completely closing
the nasal passages. The patient’s mouth
was constantly filled with saliva, and ton-
sils enormously enlarged, filling the back
part of the mouth and throat. Dr. Kegley
cocainized the tonsils and removed them
both at one sitting by means of the gal-
vano-cautery snare, the patient having
experienced no pain whatever. He saw
the .patient the next day, who said the
trouble had ceased almost simultaneously
with the removal of the tonsils. He has
had no further trouble.
Third Day—Morning Session.
Dr. A. G. Hobbs, of Atlanta, Georgia,
read a paper on “ The Surgery of Gum-
matous Growths of the Nasal ’Cavities,”
and reported four cases. In the first case
he cocainized the nasal passages with a 5
per centum spray, then attempted to intro-
duce a Blake’s snare, but found it impossi-
ble to engage the tumor in the loop. He
therefore introduced a cutting spoon, and
several large masses of the growth were cut
away. Four more sittings, at intervals of
three days, enabled him to remove the
growth entirely. The patient has com-
pletely regained her health.
Afternoon Session.
Dr. A. B. Thrasher, of Cincinnati, read
a paper on “Surgical Treatment of Nasal
Catarrh,” in which he said that there are
forms of nasal catarrh that can only be
benefited, and not cured, by medicinal
treatment. When we have mechanical ob-
structions to the lumen of the naris of a
hypertrophic, hyperplastic, or neoplastic
character, surgery offers the most direct
path for the removal of the difficulty.
What is the best method of procedure?
There are cases in which the application
of a chemical caustic answers the purpose
well. Woakes’ gouge or plow will do the
work, and by the aid of cocaine will do it
rapidly and painlessly.
In uncomplicated cases of chronic hyper-
trophic rhinitis, the conditions to be met
are:	(a) Obstructions to lumen of the
naris, and (£) abnormal condition of mu-
cous glands. The causal indications would
at once suggest removal of the redundant
tissue. The means employed should look
to the accomplishment of this end, and
with the least resulting scar-surface, so that
the natural condition of the epithelial cov-
ering might be as nearly as possible simu-
lated. To reach this result he had met
with the most marked success in the use of
the galvano-cautery knife. He first anaes-
thetizes the part with cocaine, then intro-
duces the knife to the posterior part of the
hypertrophy, turns the sharp edge toward
the tissue to be cut, heats the knife white
hot, and cuts deeply, drawing the knife
forward. .The result is a deep linear scar
in an antero-posterior direction through
the hypertrophied tissue. He cuts deeply
enough to destroy a portion of the submu-
cous erectile tissue.
After electing officers and selecting Chi-
gago as the next place of meeting, in Sep-
tember, 1889, the Association adjourned.
				

